# Erwerbsarmut und subjektive Gesundheit während der COVID-19-Pandemie: Eine Zeitvergleichsstudie mit Daten des Sozio-oekonomischen Panels 1995–2021

**DOI:** 10.1007/s00103-023-03734-z

**Published:** 2023-06-21

**Authors:** Timo-Kolja Pförtner, Ibrahim Demirer

**Affiliations:** 1 Arbeitsbereich Forschungsmethoden, Humanwissenschaftliche Fakultät, Universität zu Köln, Köln, Deutschland; 2 Institut für Medizinsoziologie, Versorgungsforschung und Rehabilitationswissenschaft, Medizinische und Humanwissenschaftliche Fakultät, Universität zu Köln, Köln, Deutschland

**Keywords:** Erwerbsarmut, Prekäre Beschäftigung, Subjektive Gesundheit, COVID-19, Trend, SOEP, Working poverty, Precarious employment, Self-rated health, COVID-19, Trend, SOEP

## Abstract

**Hintergrund:**

Erwerbsarme gelten als vulnerable Gruppe. Die vorliegende Studie untersucht, ob sich die Gesundheitsunterschiede zwischen Erwerbsarmen und Nicht-Erwerbsarmen während der COVID-19-Pandemie verstärkt haben, und führt dazu einen Zeitvergleich mit früheren Phasen ökonomischer Krisen und arbeitsmarktpolitischer Reformen durch.

**Methoden:**

Die Analysen basieren auf dem Sozio-ökonomischen Panel (SOEP, 1995–2020) und der Sondererhebung zu den sozioökonomischen Faktoren und Folgen der Verbreitung des Coronavirus in Deutschland (SOEP-CoV, 2020–2021). Alle Erwerbstätigen im Alter von 18–67 Jahren wurden in den Analysen berücksichtigt, um die Risiken einer schlechten subjektiven Gesundheit durch Erwerbsarmut auf Basis gepoolter logistischer Regression nach Geschlecht zu berechnen.

**Ergebnisse:**

Die subjektive Gesundheit verbesserte sich im Allgemeinen in der COVID-19-Pandemie. Die Unterschiede im Gesundheitszustand blieben zwischen Erwerbsarmen und Nicht-Erwerbsarmen zwischen 1995 und 2021 relativ konstant. Personen, die im Zeitverlauf häufiger von Erwerbsarmut betroffen waren, wiesen das höchste Risiko einer unzureichenden Gesundheit auf. Die mit der Häufigkeit von Erwerbsarmut assoziierten Gesundheitsunterschiede sind im Zeitverlauf angestiegen und erreichten bei beiden Geschlechtern in der Pandemie ihren Höhepunkt. Signifikante Geschlechtsunterschiede konnten nicht identifiziert werden.

**Diskussion:**

Die Studie verdeutlicht die gesellschaftliche Verankerung von Erwerbsarmut als Determinante einer unzureichenden Gesundheit. Insbesondere Personen, die im Erwerbsleben häufiger von Erwerbsarmut betroffen waren, gelten als besonders vulnerabel gegenüber einer unzureichenden Gesundheit. Tendenziell erscheint die COVID-19-Pandemie diesen Gradienten in der Gesundheit zu verstärken.

## Abstract (en)

**Background:**

The working poor are considered a vulnerable group. This study examines whether health disparities between working-poor and non-working-poor workers have worsened in the wake of the COVID-19 pandemic by comparing them over time with earlier periods of economic crisis and social and labor market policy reform.

**Methods:**

The analyses are based on the Socioeconomic Panel (SOEP, 1995–2020) and the Special Survey on Socioeconomic Factors and Consequences of the Spread of Coronavirus in Germany (SOEP-CoV, 2020–2021). All employed persons aged 18–67 years were considered for the analyses to calculate the risks of poor subjective health due to working poverty using pooled logistic regression by sex.

**Results:**

Subjective health improved during the COVID-19 pandemic. Differences in health between the working poor and those who were not working poor remained relatively constant between 1995 and 2021. Individuals who were more likely to be in working poverty over time had the highest risk of inadequate health. The health disparities associated with the frequency of working poverty increased over time and peaked for both sexes in the pandemic. Significant sex differences were not identified.

**Discussion:**

This study illustrates the social embeddedness of working poverty as a determinant of poor health. In particular, those who were more likely to experience working poverty during their working lives are considered to be particularly vulnerable to inadequate health. Tendentially, the COVID-19 pandemic appears to reinforce this gradient in health.

## Hintergrund

Die Erwerbsarbeit besitzt für die Lebensgestaltung und Identitätsentwicklung nach wie vor einen hohen Stellenwert. Sie ist Basis materiellen Wohlstands und gilt als Quelle für Anerkennung, soziale Integration und Prestige [1]. Seit den Arbeitsmarktreformen im Zuge der Agenda 2010 und dem Erstarken des Niedriglohnsektors in Deutschland hat die gesellschaftspolitische Debatte um eine gerechte und existenzsichernde Entlohnung von Arbeit stark zugenommen [2–4]. Ein besonderer Fokus liegt hierbei auf Menschen, die trotz ihrer Erwerbstätigkeit armutsgefährdet sind. Als armutsgefährdet gelten Erwerbstätige, wenn das ihnen zur Verfügung stehende Haushaltseinkommen nach Berücksichtigung von Steuern und Sozialtransferleistungen weniger als 60 % des bundesdeutschen Nettodurchschnittseinkommens (Median) beträgt [5]. Die sogenannte Erwerbsarmut ist einerseits Ausdruck einer benachteiligten Stellung am Arbeitsmarkt, die mit beruflichen Instabilitäten, einer geringeren sozialrechtlichen (Ab‑)Sicherung, einer unzureichenden Integration in den Arbeitsmarkt und fehlenden Möglichkeiten der Weiterbildung einhergehen kann [6]. Andererseits charakterisiert sie einen Mangel an finanziellen Ressourcen zur Sicherung eines Lebensstandards, der es den Mitgliedern eines Haushaltes erlaubt, an der Gesellschaft teilzuhaben [1, 5, 7].

Erwerbsarmut bezieht sich damit nicht nur auf eine materielle Unterversorgung und finanzielle Unsicherheit, sondern auch auf die fehlende Möglichkeit der sozialen und kulturellen Teilhabe, wie beispielsweise Mitglied in einem Verein zu sein oder regelmäßig ein Restaurant oder Kulturveranstaltungen zu besuchen. Aufgrund ihrer benachteiligten Stellung am Arbeitsmarkt und in der Gesellschaft gelten Erwerbsarme aus gesundheitswissenschaftlicher Perspektive auch als vulnerable Gruppe.

### Der Zusammenhang zwischen Erwerbsarmut und Gesundheit

Nationale und internationale Studien zeigen, dass Erwerbsarme ein höheres Risiko für gesundheitliche Beschwerden besitzen als Erwerbstätige, die nicht armutsgefährdet sind [6, 8–10]. Welche Mechanismen den Zusammenhang zwischen Erwerbsarmut und Gesundheit erklären, haben Pförtner und Demirer erstmals untersucht [11]. Sie zeigen, dass v. a. ein unzureichender Lebensstandard sowie Sorgen um die eigene finanzielle Sicherheit die Beziehung zwischen Erwerbsarmut und Gesundheit vermitteln und weniger arbeitsbezogene Aspekte wie eine Gratifikationskrise oder Arbeitsplatzunsicherheit. Daneben wird angenommen, dass Erwerbsarme eher Tätigkeiten ausüben, die durch Instabilität, mangelnde soziale Absicherung wie auch geringe Möglichkeiten der Förderung und des Aufstiegs gekennzeichnet sind [12–14]. Armutsgefährdung kann darüber hinaus von Empfindungen der Scham, Ausgrenzung und relativen Benachteiligung begleitet werden [15]. Sehen sich Erwerbstätige fortgesetzt der Armutsgefährdung ausgesetzt, können sich Benachteiligungen über die Zeit anhäufen und zu einem gesundheitlichen Verschleiß führen, der den Ausstieg aus der Erwerbsarmut erschwert [1, 8]. Kennzeichen hierfür sind Tendenzen einer zunehmenden Polarisierung in der Gesundheit zum Nachteil Erwerbsarmer [6, 8]. Insbesondere für Männer kann die Erwerbsarmut zusätzlich eine psychosoziale Belastung sein, da sie aufgrund der geringen Entlohnung das Rollenbild eines männlichen Alleinernährers nicht erfüllen können und vom – bei Männern dominierenden – Normalarbeitsverhältnis abweichen [16, 17].

### Der Einfluss der COVID-19-Pandemie auf die Gesundheit von Erwerbsarmen

Als vulnerable Gruppe sind Erwerbsarme durch Umbrüche oder Krisen wie arbeitsmarktpolitische Reformen oder ökonomische Krisen in besonderer Weise betroffen. Diese Krisen bedürfen einer Anpassung des Individuums an veränderte Bedingungen, wie die jüngsten Krisen in Deutschland verdeutlichen. Stehen hierzu weniger Ressourcen zur Verfügung, wie es bei Erwerbsarmen häufig der Fall ist, kann dies zu gesundheitlichen Beschwerden führen [18]. Erste Studien konnten für Deutschland Indizien dafür liefern, dass während der Hartz-IV-Reformen und der Weltwirtschaftskrise 2007 die gesundheitlichen Belastungen von Erwerbsarmen zugenommen haben [9, 19]. Diese Ergebnisse reihen sich in internationale Studienergebnisse ein, die einen Anstieg gesundheitlicher Ungleichheit in Zeiten ökonomischer Krisen und arbeitsmarkt- und sozialpolitischer Reformen der Deregulierung und einer strengen Sparpolitik beobachten konnten [20–22].

Die COVID-19-Pandemie stellt ebenfalls eine Zeit des Umbruchs dar, die neben tiefgreifenden gesundheitlichen Auswirkungen auch mit wirtschaftlichen und sozialen Einschnitten einherging. Diese hatten ihren Ursprung in weltwirtschaftlichen Versorgungsunsicherheiten, in infektionsbedingten Personalausfällen wie auch in den implementierten nicht-pharmazeutischen Maßnahmen zur Eindämmung der COVID-19-Pandemie (wie beispielsweise Schließungen von Geschäften, Restaurants und Schulen, Kontaktbeschränkungen oder Grenzschließungen). Zwar konnte die Wirtschaft unter Hinzunahme sogenannter interner Flexibilitätsinstrumente – wie Kurzarbeit, Abbau von Überstunden, Anordnung von Urlaub oder Verkürzung der Arbeitszeit – auf die spezifischen Anforderungen der Pandemie reagieren [23]. Erwerbsarme sahen sich jedoch aufgrund von Nachfrage- und Einkommensausfällen, einer (möglichen) Nichtweiterbeschäftigung, einer erhöhten Infektionsgefahr, fehlenden Kinderbetreuungsangeboten wie auch mangelnden Homeoffice-Optionen besonders von der Pandemie bedroht [23–27]. So beobachtet beispielsweise Hövermann, dass untere Einkommensgruppen häufiger von coronabedingten wirtschaftlichen und finanziellen Sorgen sowie von Einbußen in der Arbeitssituation und im Einkommen berichten als höhere Einkommensgruppen [28].

### Ziel der vorliegenden Studie

Inwieweit Erwerbsarme während der COVID-19-Pandemie Einbußen in Bezug auf ihren allgemeinen Gesundheitszustand hinnehmen mussten, wurde bislang noch nicht untersucht. Eine Studie für Deutschland deutet darauf dahin, dass sich die gesundheitliche Ungleichheit in der COVID-19-Pandemie zuungunsten sozial Benachteiligter – gemessen über Bildung, Einkommen und/oder Beruf – vergrößert hat [29]. Eine andere Untersuchung konnte hingegen keine statistisch signifikanten Veränderungen im Ausmaß gesundheitlicher Ungleichheit finden [30]. Die vorliegende Studie knüpft hieran an und untersucht, ob und inwieweit sich der Zusammenhang zwischen Erwerbsarmut und subjektiver Gesundheit während der COVID-19-Pandemie in Deutschland verändert hat. Hierzu wird eine Zeitverlaufsperspektive gewählt, die den Zusammenhang zwischen Erwerbsarmut und Gesundheit während der COVID-19-Pandemie mit anderen Phasen wirtschaftlicher Krisen und arbeitsmarktpolitischer Reformen vergleicht (Tab. 1). Die Datenbasis bilden das Sozio-oekonomische Panel (SOEP) aus den Jahren 1995 bis 2020 und die dazugehörige Sondererhebung zu den sozioökonomischen Faktoren und Folgen der Verbreitung des Coronavirus in Deutschland (SOEP-CoV) aus den Jahren 2020 bis 2021.

**Table 1:** 

Phase	Beschreibung
Deregulierung (1995–1997)	Die Phase der Deregulierung ist von einer hohen Anzahl von (Langzeit‑)Arbeitslosen geprägt. In ihr wurden erste Maßnahmen der Deregulierung mit Bezug auf den Kündigungsschutz und die Anstellung befristet Beschäftigter vollzogen. Sie beinhaltet zudem Maßnahmen zur Einführung eines Mindestlohns im Bauhauptgewerbe, im Dachdeckerhandwerk und im Elektrohandwerk
Reregulierung (1998–2001)	In der Phase der Reregulierung wurden die zwischen 1995 und 1997 getroffenen Maßnahmen zum Kündigungsschutz aufgehoben und entsprechende Deregulierungsmaßnahmen zur befristeten und geringfügigen Beschäftigung eingeführt. Durch das im Jahr 1997 beschlossene und 1998 in Kraft getretene Arbeitsförderungsreformgesetz wurden unter anderem die Zumutbarkeitsregeln und der Grad der materiellen Anreize zur Aufnahme und Beibehaltung einer Beschäftigung verstärkt sowie Regelungen zur Arbeitnehmerüberlassung dereguliert
Hartz-Reformen (2001–2005)	Die Phase der Hartz-Reformen beschreibt einen erheblichen Umbau der Arbeitsmarktpolitik und die Etablierung eines Niedriglohnsektors in Deutschland vor dem Hintergrund einer hohen Anzahl von (Langzeit‑)Arbeitslosen. Sie hatte ihren Ursprung im Job-AQTIV-Gesetz aus dem Jahr 2002, das erstmals den Grad der materiellen Anreize zur Aufnahme und Beibehaltung einer Beschäftigung erhöht hat. In dieser Phase wurden darüber hinaus unter anderem Maßnahmen zur Deregulierung der Arbeitnehmerüberlassung, der befristeten und geringfügigen Beschäftigung und des Kündigungsschutzes sowie eine Neuausrichtung bzw. Erweiterung der Handlungsspielräume der aktivierenden Arbeitsmarktpolitik durchgesetzt. Damit wurden die Zumutbarkeit und die materiellen Anreize zur Aufnahme und Beibehaltung einer Beschäftigung substanziell erhöht
Reregulierung (2006–2007)	In dieser Phase der Reregulierung sank die Arbeitslosenzahl nach Jahren der Stagnation erstmals. In ihr wurden vergleichsweise kleinere Maßnahmen umgesetzt, die die Anpassungen zur Stärkung der Beschäftigung älterer Menschen betrafen sowie Deregulierungsmaßnahmen zur Vermeidung von Arbeitslosigkeit bei saisonalen Arbeitsausfällen. Darüber hinaus wurde im Postdienstleistungssektor und im Gebäudereinigungsgewerbe ein Mindestlohn eingeführt
Weltfinanzkrise (2008–2009)	Die Phase der Weltfinanzkrise beschreibt eine Zeit der ökonomischen Unsicherheit, die insbesondere im internationalen Raum mit erheblichen wirtschaftlichen Einschnitten einherging und in Deutschland auch aufgrund der politischen Unterstützung bei der Förderung des Kurzarbeitergelds relativ gut bewältigt werden konnte. In dieser Zeit wurden unter anderem die Zumutbarkeitsregeln und der Grad der materiellen Anreize zur Aufnahme einer Beschäftigung für ältere Menschen erhöht, einige Reregulierungsmaßnahmen und Anpassungen der aktiven Arbeitsförderung getroffen sowie Reregulierungsmaßnahmen zu den Entsenderichtlinien von Arbeitnehmer*innen durchgesetzt
Post-Rezession (2010–2020)^a^	Die Phase der Post-Rezession ist von einem allgemein hohen Beschäftigungsniveau geprägt. In ihr wurden unter anderem verschiedene Reregulierungsmaßnahmen zum Schutz von Arbeitsnehmer*innen vor Arbeitslosigkeit (Stichwort: Kurzarbeit) und zur Integration von Migrant*innen und Langzeitarbeitslosen in den Arbeitsmarkt getroffen. Auch wurden die Arbeitsbedingungen von Leiharbeiter*innen und geringfügig Beschäftigten mittels Reregulierungsmaßnahmen gestärkt sowie im Jahr 2015 ein allgemeiner Mindestlohn eingeführt, der im zeitlichen Verlauf weiter angepasst wurde
COVID-19-Pandemie (2020–2021)^b^	Die Phase der COVID-19-Pandemie ging mit erheblichen gesundheitlichen, sozialen und wirtschaftlichen Einschnitten einher. Diese hatten ihren Ursprung im Infektionsgeschehen und den damit assoziierten weltwirtschaftlichen Versorgungsunsicherheiten, infektionsbedingten Personalausfällen und implementierten nicht-pharmazeutischen Maßnahmen zur Eindämmung des Infektionsgeschehens. Die Wirtschaft konnte auf diese bis zu einem gewissen Grad durch Hinzunahme interner Flexibilitätsinstrumente und politischer Unterstützungsmaßnahmen reagieren. Auch wurden verschiedene staatliche Unterstützungsmöglichkeiten für spezifische Gruppen wie Familien, Kinder oder Arbeitslose eingeführt, um insbesondere den finanziellen Herausforderungen der Pandemie zu begegnen

^a^Angaben, die zwischen 2010 und Februar 2020 erfasst wurden, wurden der Post-Rezessionsphase zugeordnet

^b^Die Phase der COVID-19 Pandemie schließt alle Angaben ein, die seit März 2020 erhoben wurden

## Methoden

Die empirischen Analysen basieren auf den Daten des SOEP aus den Jahren 1995 bis 2020 und des SOEP-CoV aus den Jahren 2020 und 2021. Das SOEP ist eine wissenschaftsgetragene, für Deutschland repräsentative Längsschnittbefragung, bei der seit 1984 im jährlichen Turnus aus repräsentativ ausgewählten Haushalten alle Personen ab einem Alter von 17 Jahren persönlich befragt werden [31, 32]. Das SOEP erfasst objektive wie subjektive Indikatoren aus der soziologischen, psychologischen, politikwissenschaftlichen und gesundheitswissenschaftlichen Forschung, um die Stabilität und den Wandel der Lebensbedingungen der in Deutschland lebenden Menschen zu erfassen. Befragungsteilnehmer*innen des SOEP werden über den Haushalt ermittelt, welcher größtenteils über ein mehrstufiges Stichprobenverfahren (meist per Random-Walk) gezogen wird. Erfasst werden die Information der Panelteilnehmer*innen im SOEP entweder durch ein persönlich-mündliches Interview, das mittels Papierfragebogen (PAPI) oder computerunterstützt mit dem Laptop (CAPI) durchgeführt wird, oder durch das Selbstausfüllen des Fragebogens durch die befragte Person, entweder im Rahmen einer interviewerbetreuten Befragung (telefonisch oder vor Ort) oder rein schriftlich [33]. Um demografischen Veränderungen und dem Ausscheiden von Panelteilnehmer*innen im SOEP zu begegnen, wurde die Stichprobe seit 1984 um weitere Teilstichproben ergänzt. In der aktuellen Version setzt sich das SOEP aus verschiedenen Teilstichproben zusammen [34]. Aus diesen nehmen im Durchschnitt ca. 86,0 % der Personen an der Befragung im SOEP teil, wobei die Teilnahmequote zwischen den einzelnen Stichproben und Erhebungswellen zwischen 61,2 % und 96,7 % stark variiert.

Zusätzlich zum SOEP berücksichtigt die vorliegende Studie Informationen aus den Sondererhebungen des SOEP-CoV aus den Jahren 2020 und 2021. Ziel der Sondererhebungen war es, die akuten, mittelfristigen und langfristigen sozioökonomischen Folgen der Verbreitung des Coronavirus in Deutschland zu erfassen [35]. Dazu wurden Panelteilnehmer*innen aus dem SOEP zu 2 Zeitpunkten (April bis Juli 2020 sowie Januar und Februar 2021) innerhalb der COVID-19-Pandemie befragt [36]. Im Gegensatz zur üblichen SOEP-Befragung wurden in SOEP-CoV nur eine Person je Haushalt stellvertretend für alle Haushaltsmitglieder befragt. In der ersten Welle der SOEP-CoV-Sondererhebung wurden 12.000 Personen aus der SOEP-Stichprobe zur Befragung eingeladen. Von diesen nahmen 55,8 % (*n* = 6694) teil. Bei der zweiten SOEP-CoV-Sondererhebung wurden die Personen, die an der ersten Welle teilgenommen hatten, zu einer weiteren Befragung eingeladen. Von diesen nahmen 90,2 % erneut teil (*n* = 6038). Sowohl die erste als auch die zweite Befragung wurden ausschließlich mittels computergestützter telefonischer Interviews (CATI) durchgeführt [37].

**Figure 1:**
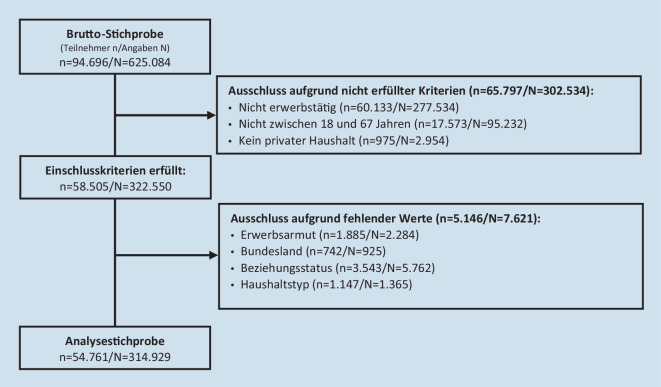

Die vorliegenden Analysen basieren auf allen Befragungspersonen, die zum Zeitpunkt der Erhebung zwischen 18 und 67 Jahre alt waren, einer Erwerbstätigkeit nachgingen (Vollzeit, Teilzeit oder geringfügig), in einem Privathaushalt lebten und für die keine fehlenden Werte vorlagen (Abb. 1). Von den insgesamt 94.696 erfüllten 65.797 Befragungspersonen mindestens zu einem Befragungszeitpunkt nicht die Einschlusskriterien, so dass in der Summe 302.534 Angaben aus den Analysen ausgeschlossen werden musste. Darüber hinaus lagen bei 5146 Personen mindestens zu einem Erhebungszeitpunkt in den berücksichtigten Variablen fehlende Werte vor, so dass weitere 7621 Angaben nicht in den Analysen berücksichtigt werden konnten. Insgesamt lagen damit 314.929 Angaben (Beobachtungen) von insgesamt 54.761 Personen vor. Im Mittel nahmen die Befragungspersonen an 5,51 von 28 möglichen Erhebungswellen teil. Erwerbsarme nahmen im Mittel weniger häufig an der Befragung teil (4,43 Erhebungswellen) als Nicht-Erwerbsarme (6,03 Erhebungswellen). Die Teilnahmequote an den Erhebungen war höher für Erwerbstätige, die im Beobachtungszeitraum nie erwerbsarm waren (5,47 Erhebungswellen), als für Erwerbstätige, die im Beobachtungszeitraum einmal armutsgefährdet waren (5,04 Erhebungswellen). Erwerbstätige, die im Beobachtungszeitraum zweimal (6,44 Erhebungswellen), dreimal (7,69 Erhebungswellen) oder mindestens viermal armutsgefährdet waren (10,69 Erhebungswellen), nahmen häufiger an der Befragung teil.

Die Gesundheit einer Person wurde in der vorliegenden Studie über einen Indikator zur subjektiven Einschätzung des eigenen Gesundheitszustandes gemessen. Die subjektive Gesundheit wird im SOEP in Anlehnung an die Empfehlungen der Weltgesundheitsorganisation (WHO; [38]) und der EURO-REVES-2-Gruppe [39] mit: „Wie würden Sie Ihren gegenwärtigen Gesundheitszustand beschreiben?“, auf einer 5‑stufigen Likert-Skala („sehr gut“, „gut“, „zufriedenstellend“, „weniger gut“ und „schlecht“) erfasst. Für die vorliegende Auswertung wurde die Variable dichotomisiert, und zwar in Erwerbstätige, die von einem mindestens zufriedenstellenden Gesundheitszustand berichteten (mit der Ausprägung 0), und solchen, die von einem weniger guten bis schlechten Gesundheitszustand berichteten (mit der Ausprägung 1).

Die Messung von Erwerbsarmut basiert im SOEP auf einer offenen Frage zum verfügbaren Haushaltseinkommen im letzten Monat nach Berücksichtigung von Steuerabzügen und Transferleistungen. Der gängigen Praxis folgend, haben wir das verfügbare Haushaltseinkommen um die Haushaltsgröße und -zusammensetzung mithilfe der modifizierten Skala der Organisation für wirtschaftliche Zusammenarbeit und Entwicklung (OECD) angepasst, so dass jedes Mitglied eines Haushalts ein nettoäquivalentgewichtetes Einkommen erhält [40]. Eine erwerbstätige Person wurde zum Zeitpunkt der Erhebung als armutsgefährdet beziehungsweise erwerbsarm definiert, wenn ihr Nettoäquivalenzeinkommen weniger als 60 % des bundesdeutschen Medianeinkommens betrug [7]. Auf Basis der zur Verfügung stehenden Informationen zur Erwerbsarmut wurde darüber hinaus ein Indikator gebildet, der die Häufigkeit von Erwerbsarmut im gesamten Beobachtungszeitraum erfasst. Dieser unterscheidet zwischen Beschäftigten, die im gesamten Beobachtungszeitraum nie, ein-, zwei- bis drei- oder viermal oder häufiger erwerbsarm waren.

Als Kontrollvariablen wurden das Alter, das Bundesland, der Beziehungsstatus und der Haushaltstyp berücksichtigt. Schließlich wurden in Anlehnung an Pförtner et al. [9] die einzelnen Erhebungswellen des SOEP und des SOEP-CoV unterschiedlichen Phasen ökonomischer Krisen und arbeitsmarktpolitischer Reformen zugeordnet und um die Phase der COVID-19-Pandemie erweitert (Tab. 1). Die Angaben der Befragungspersonen wurden nach Maßgabe des Interviewzeitpunktes den unterschiedlichen Phasen zugeordnet. Die Phase der COVID-19 Pandemie schließt alle Angaben ein, die seit März 2020 erhoben wurden. Angaben, die zwischen 2010 und Februar 2020 erfasst wurden, wurden der Post-Rezessionsphase zugeordnet. Die Phasen berücksichtigen Maßnahmen der Regulierung und Deregulierung zum Mindestlohn, zur geringfügigen, kurzfristigen und befristeten Beschäftigung, zur Arbeitnehmerüberlassung, zur Arbeitslosigkeit und zum Kündigungsschutz, da diese direkten wie indirekten Einfluss auf die Lage Erwerbsarmer nehmen [41]. Eine umfassende Übersicht zu den in Deutschland implementierten Reformen liefert Steffen [42].

Zu Beginn der statistischen Analysen wurde zunächst der Anteil an Beschäftigten erfasst, die von einem unzureichenden Gesundheitszustand berichtet haben, sowie der Anteil an Beschäftigten, die erwerbsarm waren. Um signifikante Unterschiede zwischen den einzelnen Zeitperioden zu identifizieren, wurden univariate logistische Regressionsmodelle mit einer Trendvariable (Referenz: COVID-19-Pandemie) und panel-robusten Standardfehlern berechnet.

Im Anschluss wurden die Zeitverlaufsunterschiede der Beziehung zwischen subjektiver Gesundheit und Erwerbsarmut in 2 Schritten untersucht. In einem ersten Schritt wurde eine gepoolte logistische Regression für eine schlechte subjektive Gesundheit nach Erwerbsarmut zum Zeitpunkt der Erhebung und nach der Häufigkeit von Erwerbsarmut im Beobachtungszeitraum unter Hinzunahme der Kontrollvariablen berechnet. Um signifikante Unterschiede zwischen den einzelnen Zeitphasen zu identifizieren, wurde die Regression um eine Interaktionsvariable ergänzt. Diese Variable bildet die Interaktion zwischen den einzelnen Zeitphasen und den beiden Indikatoren von Erwerbsarmut ab, bei der die Phase der COVID-19-Pandemie die Referenzkategorie darstellt. Die Schätzungen basierten auf panel-robusten Standardfehlern, um die Abhängigkeit in der längsschnittlichen Datenstruktur empirisch zu berücksichtigen. In einem zweiten Schritt wurden auf Basis der Schätzungen aus den gepoolten logistischen Regressionsmodellen eine unzureichende Gesundheit nach Erwerbsarmut zum Zeitpunkt der Erhebung und nach der Häufigkeit von Erwerbsarmut für die jeweiligen Phasen als „adjusted predictions at representative values“ (APR) vorhergesagt und grafisch dargestellt. Alle Analysen wurden mit Stata, Version 16.1 (StataCorp LP, College Station, Texas) durchgeführt.

## Ergebnisse

Tab. 2 weist die Stichprobenzusammensetzung und die fehlenden Werte in den einzelnen Zeitphasen entlang zentraler Untersuchungsmerkmale aus. Die Stichprobenzusammensetzung mit Bezug auf Geschlecht, Bundesland, Beziehungsstatus und Häufigkeit von Erwerbsarmut unterscheidet sich in der Phase der COVID-19-Pandemie nur marginal von den anderen Phasen. Die Stichprobe der COVID-19-Pandemie setzt sich im Vergleich zu den anderen Phasen häufiger aus Personen zusammen, die in einem Einpersonenhaushalt leben. Personen, die in einem Haushalt mit Beziehungspartner mit Kindern leben, sind hingegen in der Phase der Pandemie weniger häufig vertreten als in den Erhebungen zuvor. Darüber hinaus schätzen die Befragungsteilnehmer*innen ihren subjektiven Gesundheitsstatus während der COVID-19-Pandemie besser ein als in den vorherigen Phasen. Die Erwerbsarmut ist während der COVID-19-Pandemie angestiegen. Mit 17,6 % bei Männern und 8,4 % bei Frauen ist der Anteil fehlender Werte in der Phase der Pandemie am höchsten.

**Table 2:** 

	Deregulierung 1995–1997				Reregulierung 1998–2001				Hartz-Reformen 2002–2005				Reregulierung 2006–2007				Weltfinanzkrise 2008–2009				Post-Rezession 2010–2020^a^				COVID-19-Pandemie 2020–2021^b^			
	Männer		Frauen		Männer		Frauen		Männer		Frauen		Männer		Frauen		Männer		Frauen		Männer		Frauen		Männer		Frauen	
*Gesamt (N/%)*	*12.682*	*100,0*	*9530*	*100,0*	*22.655*	*100,0*	*17.984*	*100,0*	*25.626*	*100,0*	*22.233*	*100,0*	*11.989*	*100,0*	*10.937*	*100,0*	*11.161*	*100,0*	*10.583*	*100,0*	*72.443*	*100,0*	*72.435*	*100,0*	*10.915*	*100,0*	*11.370*	*100,0*
Gültig	12.585	99,2	9465	99,3	22.618	99,8	17.958	99,9	25.566	99,8	22.189	99,8	11.961	99,8	10.911	99,8	11.142	99,8	10.560	99,8	69.012	95,3	71.540	98,8	8995	82,4	10.420	91,6
Fehlende Werte	97	0,8	65	0,7	37	0,2	26	0,1	60	0,2	44	0,2	28	0,2	26	0,2	19	0,2	23	0,2	3431	4,7	895	1,2	1920	17,6	950	8,4
*Bundesländer (n/%)*																												
Neue	3484	27,5	2978	31,2	5585	24,7	4954	27,5	5887	23,0	5499	24,7	2802	23,4	2761	25,2	2711	24,3	2742	25,9	15.909	22,0	16.567	22,9	2185	20,0	2289	20,1
Alte	9198	72,5	6552	68,8	17.070	75,3	13.030	72,5	19.739	77,0	16.734	75,3	9187	76,6	8176	74,8	8450	75,7	7841	74,1	56.534	78,0	55.868	77,1	8315	76,2	8571	75,4
Fehlende Werte	–	–	–	–	–	–	–	–	–	–	–	–	–	–	–	–	–	–	–	–	–	–	–	–	415	3,8	510	4,5
*Alter (n/%)*																												
18–29 Jahre	2558	20,2	2194	23,0	3559	15,7	3258	18,1	3190	12,4	3376	15,2	1551	12,9	1623	14,8	1440	12,9	1542	14,6	10.099	13,9	9346	12,9	1701	15,6	1260	11,1
30–39 Jahre	4164	32,8	2938	30,8	7432	32,8	5404	30,0	6979	27,2	5720	25,7	2823	23,5	2445	22,4	2404	21,5	2170	20,5	15.381	21,2	15.044	20,8	2447	22,4	2006	17,6
40–49 Jahre	3124	24,6	2606	27,3	6202	27,4	5446	30,3	7904	30,8	7327	33,0	3818	31,8	3661	33,5	3571	32,0	3449	32,6	21.000	29,0	22.307	30,8	2650	24,3	3281	28,9
50–67 Jahre	2836	22,4	1792	18,8	5462	24,1	3876	21,6	7553	29,5	5810	26,1	3797	31,7	3208	29,3	3746	33,6	3422	32,3	25.963	35,8	25.738	35,5	4117	37,7	4823	42,4
Fehlende Werte	–	–	–	–	–	–	–	–	–	–	–	–	–	–	–	–	–	–	–	–	–	–	–	–	–	–	–	–
*Beziehungsstatus (n/%)*																												
Verheiratet	8878	70,0	6214	65,2	15.412	68,0	11.372	63,2	17.237	67,3	14.132	63,6	7817	65,2	6699	61,3	7106	63,7	6383	60,3	44.828	61,9	41.652	57,5	5849	53,6	6403	56,3
Verpartnert	2151	17,0	1988	20,9	4076	18,0	3862	21,5	4820	18,8	4817	21,7	2438	20,3	2490	22,8	2342	21,0	2421	22,9	14.137	19,5	16.193	22,4	1996	18,3	2452	21,6
Single	1578	12,4	1280	13,4	3166	14,0	2750	15,3	3569	13,9	3284	14,8	1734	14,5	1748	16,0	1713	15,3	1779	16,8	10.285	14,2	13.927	19,2	1525	14,0	2278	20,0
Fehlende Werte	75	0,6	48	0,5	1	0,0	–	–	–	–	–	–	–	–	–	–	–	–	–	–	3193	4,4	663	0,9	1545	14,2	237	2,1
*Haushaltstyp (n/%)*																												
Eine Person	1065	8,4	837	8,8	2480	10,9	1895	10,5	3031	11,8	2268	10,2	1625	13,6	1258	11,5	1551	13,9	1277	12,1	10.031	13,8	8637	11,9	2171	19,9	1598	14,1
2 Personen	2788	22,0	2602	27,3	5369	23,7	5155	28,7	6566	25,6	6631	29,8	3183	26,5	3346	30,6	3047	27,3	3259	30,8	17.959	24,8	19.417	26,8	2408	22,1	2596	22,8
Alleinerziehend	346	2,7	678	7,1	606	2,7	1391	7,7	641	2,5	1689	7,6	289	2,4	908	8,3	290	2,6	947	8,9	2268	3,1	8963	12,4	377	3,5	1487	13,1
Paar mit Kindern	7723	60,9	4940	51,8	13.421	59,2	8956	49,8	14.774	57,7	11.104	49,9	6643	55,4	5196	47,5	6079	54,5	4897	46,3	40.068	55,3	33.537	46,3	5202	47,7	4873	42,9
Andere	760	6,0	473	5,0	779	3,4	587	3,3	601	2,3	532	2,4	239	2,0	216	2,0	188	1,7	197	1,9	1947	2,7	1718	2,4	324	3,0	274	2,4
Fehlende Werte	–	–	–	–	–	–	–	–	13	0,1	9	0,0	10	0,1	13	0,1	6	0,1	6	0,1	170	0,2	163	0,2	433	4,0	542	4,8
*Subjektive Gesundheit (n/%)*																												
Mindestens zufriedenstellend	11.446	90,3	8357	87,7	20.614	91,0	16.127	89,7	23.207	90,6	19.763	88,9	10.812	90,2	9698	88,7	9998	89,6	9300	87,9	64.990	89,7	63.129	87,2	10.248	93,9	10.333	90,9
Weniger gut/schlecht	1213	9,6	1156	12,1	2005	8,9	1831	10,2	2372	9,3	2435	11,0	1159	9,7	1226	11,2	1150	10,3	1266	12,0	7390	10,2	9251	12,8	653	6,0	1031	9,1
Fehlende Werte	23	0,2	17	0,2	36	0,2	26	0,1	47	0,2	35	0,2	18	0,2	13	0,1	13	0,1	17	0,2	63	0,1	55	0,1	14	0,1	6	0,1
*Erwerbsarmut zum Zeitpunkt der Erhebung (n/%)*																												
Nein	11.391	89,8	8615	90,4	20.753	91,6	16.410	91,2	23.814	92,9	20.320	91,4	11.049	92,2	9905	90,6	10.020	89,8	9276	87,7	61.890	85,4	62.219	85,9	8110	74,3	8932	78,6
Ja	1291	10,2	915	9,6	1902	8,4	1574	8,8	1799	7,0	1904	8,6	930	7,8	1019	9,3	1135	10,2	1301	12,3	9685	13,4	9971	13,8	2246	20,6	1883	16,6
Fehlende Werte	–	–	–	–	–	–	–	–	13	0,1	9	0,0	10	0,1	13	0,1	6	0,1	6	0,1	868	1,2	245	0,3	559	5,1	555	4,9
*Häufigkeit von Erwerbsarmut im gesamten Beobachtungszeitraum (n/%)*																												
Nie	8961	70,7	6733	70,6	15.980	70,5	12.436	69,1	18.539	72,3	15.409	69,3	8521	71,1	7417	67,8	7811	70,0	7020	66,3	48.474	66,9	47.534	65,6	6985	64,0	7495	65,9
Einmal	1489	11,7	1143	12,0	2718	12,0	2228	12,4	2906	11,3	2630	11,8	1404	11,7	1335	12,2	1330	11,9	1325	12,5	9588	13,2	9316	12,9	1632	14,9	1478	13,0
2- bis 3‑mal	1182	9,3	858	9,0	2049	9,0	1669	9,3	2169	8,5	2047	9,2	1045	8,7	1070	9,8	1039	9,3	1104	10,4	7757	10,7	8048	11,1	1304	11,9	745	6,6
4‑mal oder häufiger	1050	8,3	796	8,4	1908	8,4	1651	9,2	2009	7,8	2146	9,7	1017	8,4	1114	10,2	981	8,8	1134	10,7	6125	8,5	7434	10,3	759	7,0	1117	9,8
Fehlende Werte	–	–	–	–	–	–	–	–	3	0,0	1	0,0	2	0,0	–	–	–	–	–	–	499	0,7	103	0,1	235	2,2	510	4,5

^a^Angaben, die zwischen 2010 und Februar 2020 erfasst wurden, wurden der Post-Rezessionsphase zugeordnet

^b^Die Phase der COVID-19 Pandemie schließt alle Angaben ein, die seit März 2020 erhoben wurden

**Figure 2:**
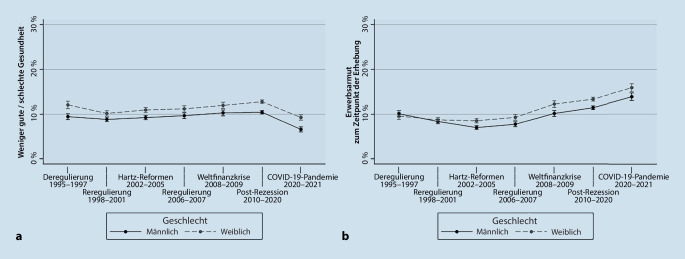

In Abb. 2a sind die Verläufe einer unzureichenden subjektiven Gesundheit über die Zeit für die Analysestichprobe dargestellt. Es wird deutlich, dass Frauen ihre Gesundheit signifikant häufiger als schlecht einschätzen als Männer und dass die selbsteingeschätzte Gesundheit über die Zeit relativ konstant verläuft, bis sie sich in der COVID-19-Pandemie im Vergleich zu den anderen Phasen signifikant (*p* < 0,05) verbessert. Die Erwerbsarmut ist im Zeitverlauf stetig gestiegen und betraf signifikant häufiger Frauen als Männer (Abb. 2b).

Tab. 3 veranschaulicht die Ergebnisse der gepoolten logistischen Regressionsanalysen für eine unzureichende subjektive Gesundheit nach Status und Häufigkeit der Erwerbsarmut getrennt nach Geschlecht.

**Table 3:** 

	Deregulierung 1995–1997		Reregulierung 1998–2001		Hartz-Reformen 2002–2005		Reregulierung 2006–2007		Weltfinanzkrise 2008–2009		Post-Rezession 2010–2020^a^		COVID-19-Pandemie 2020–2021^b^	
	OR (95 % KI)	*p*-Wert^c^	OR (95 % KI)	*p*-Wert^c^	OR (95 % KI)	*p*-Wert^c^	OR (95 % KI)	*p*-Wert^c^	OR (95 % KI)	*p*-Wert^c^	OR (95 % KI)	*p*-Wert^c^	OR (95 % KI)	*p*-Wert^c^
**Männer (*n*** **=** **28.134/*N*** **=** **161.879)**^**d**^														
Erwerbsarm zum Zeitpunkt der Erhebung	1,569*** (1,256–1,960)	*p* = 0,970	1,377** (1,145–1,656)	*p* = 0,405	1,600*** (1,341–1,909)	*p* = 0,862	1,475** (1,163–1,870)	*p* = 0,737	1,716*** (1,389–2,120)	*p* = 0,541	1,756*** (1,594–1,934)	*p* = 0,321	1,559*** (1,241–1,959)	*Referenz*
Pseudo R^2^	0,0494	–	0,0328	–	0,0313	–	0,0321	–	0,0351	–	0,0299	–	0,0306	–
*Häufigkeit von Erwerbsarmut im Beobachtungszeitraum (Referenz: nie)*														
1‑mal	1,021 (0,790–1,319)	*p* = 0,144	1,285** (1,068–1,546)	*p* = 0,776	1,271* (1,050–1,540)	*p* = 0,728	1,158 (0,924–1,452)	*p* = 0,395	1,406** (1,133–1,745)	*p* = 0,807	1,466*** (1,300–1,654)	*p* = 0,532	1,347* (1,026–1,770)	*Referenz*
2- bis 3‑mal	1,691*** (1,307–2,189)	*p* = 0,993	1,299* (1,043–1,617)	*p* = 0,163	1,585*** (1,289–1,948)	*p* = 0,733	1,314* (1,015–1,700)	*p* = 0,207	1,369* (1,066–1,758)	*p* = 0,281	1,555*** (1,370–1,765)	*p* = 0,584	1,688*** (1,254–2,274)	
4‑mal oder häufiger	1,244 (0,889–1,744)	*p* = 0,053	1,460** (1,158–1,840)	*p* = 0,144	1,650*** (1,330–2,048)	*p* = 0,394	1,495** (1,172–1,907)	*p* = 0,182	1,589*** (1,244–2,031)	*p* = 0,309	1,700*** (1,471–1,964)	*p* = 0,386	1,938*** (1,420–2,645)	
Pseudo R^2^	0,0503	–	0,0340	–	0,0335	–	0,0329	–	0,0352	–	0,0311	–	0,0341	–
**Frauen (*n*** **=** **26.631/*N*** **=** **153.043)**^**a**^														
Erwerbsarm zum Zeitpunkt der Erhebung	1,362** (1,088–1,705)	*p* = 0,143	1,324** (1,095–1,601)	*p* = 0,068	1,462*** (1,242–1,721)	*p* = 0,244	1,327** (1,073–1,642)	*p* = 0,089	1,484*** (1,235–1,783)	*p* = 0,323	1,624*** (1,498–1,761)	*p* = 0,689	1,687*** (1,413–2,014)	*Referenz*
Pseudo R^2^	0,0344	–	0,0336	–	0,036	–	0,0278	–	0,0221	–	0,0208	–	0,0133	–
*Häufigkeit von Erwerbsarmut im Beobachtungszeitraum (Referenz: nie)*														
1‑mal	1,334* (1,049–1,696)	*p* = 0,838	1,295** (1,068–1,569)	*p* = 0,660	1,296**	*p* = 0,658	1,053	*p* = 0,079	1,194	*p* = 0,330	1,249***	*p* = 0,362	1,379**	*Referenz*
					(1,083–1,552)		(0,842–1,317)		(0,971–1,468)		(1,121–1,393)		(1,117–1,702)	
2- bis 3‑mal	1,357* (1,047–1,758)	*p* = 0,889	1,499*** (1,219–1,843)	*p* = 0,635	1,591*** (1,318–1,921)	*p* = 0,369	1,648*** (1,317–2,061)	*p* = 0,297	1,374** (1,101–1,714)	*p* = 0,939	1,457*** (1,306–1,624)	*p* = 0,701	1,391** (1,101–1,756)	
4‑mal oder häufiger	1,034 (0,760–1,407)	*p* = 0,018	1,170 (0,920–1,489)	*p* = 0,046	1,418*** (1,167–1,724)	*p* = 0,339	1,576*** (1,268–1,961)	*p* = 0,806	1,359** (1,095–1,685)	*p* = 0,227	1,594*** (1,420–1,789)	*p* = 0,809	1,639*** (1,305–2,058)	*–*
Pseudo R^2^	0,0352	–	0,0352	–	0,0257	–	0,0319	–	0,0219	–	0,0209	–	0,0123	*–*
*KI* Konfidenzintervall, *OR* Odds Ratio *p < 0,05 **p < 0,01 ***p < 0,000 ^a^Angaben, die zwischen 2010 und Februar 2020 erfasst wurden, wurden der Post-Rezessionsphase zugeordnet ^b^Die Phase der COVID-19 Pandemie schließt alle Angaben ein, die seit März 2020 erhoben wurden ^c^Der *p*-Wert gibt an, ob sich der Zusammenhang zwischen Erwerbsarmut und subjektiver Gesundheit in der berücksichtigten Zeitphase von der Phase der COVID-19-Pandemie (Referenzkategorie) signifikant unterscheidet ^d^Die Ergebnisse basieren auf gepoolten Regressionsmodellen mit geclusterten Standardfehlern unter Kontrolle von Bundesland, Alter, Haushaltstyp und Beziehungsstatus														

**Figure 3:**
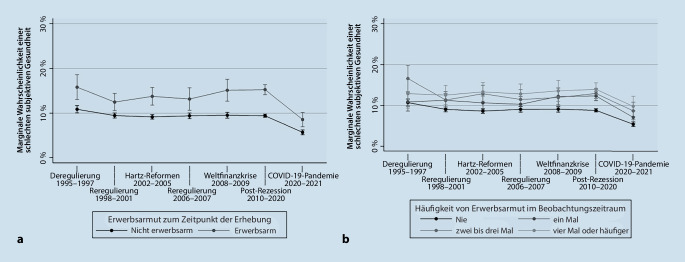

Für Männer zeigt sich, dass die Erwerbsarmut in allen Zeitphasen signifikant mit einer unzureichenden subjektiven Gesundheit assoziiert war. Im Vergleich zu den 2 vorangegangen Zeitphasen der Weltwirtschaftskrise und Post-Rezession war der Zusammenhang zwischen Erwerbsarmut und Gesundheit während der Pandemie leicht schwächer, wobei die Assoziation zwischen Erwerbsarmut und subjektiver Gesundheit nicht signifikant zwischen den jeweiligen Zeitphasen variierte. Abb. 3a verdeutlicht einen vom Armutsstatus unabhängigen Rückgang des unzureichenden Gesundheitszustandes während der Pandemie. Dieser Rückgang verläuft für Erwerbsarme leicht stärker als für Nicht-Erwerbsarme. Die Häufigkeit von Erwerbsarmut ist in der ersten Zeitphase der Deregulierung (1995–1997) bei Männern nicht deutlich mit einer unzureichenden subjektiven Gesundheit assoziiert (Tab. 3). Im Zeitverlauf nimmt die Beziehung zwischen subjektiver Gesundheit und Häufigkeit von Erwerbsarmut stetig zu und resultiert in einem Gradienten in der subjektiven Gesundheit zuungunsten jener Männer, die im Beobachtungszeitraum häufiger von Erwerbsarmut betroffen waren. Dieser Gradient in der Gesundheit war in der Phase der COVID-19-Pandemie (2020–2021) am stärksten ausgeprägt. Abb. 3b zeigt, dass in der COVID-19-Pandemie unabhängig von der Häufigkeit von Erwerbsarmut die Wahrscheinlichkeit eines unzureichenden Gesundheitszustandes im Vergleich zu den vorangegangenen Phasen zurückging. Auch wird ersichtlich, dass sich die absoluten Unterschiede im Gesundheitszustand insbesondere zwischen den Personen, die im Beobachtungszeitrum nie erwerbsarm waren, und denjenigen, die im Beobachtungszeitraum einmal erwerbsarm waren, angenähert haben.

**Figure 4:**
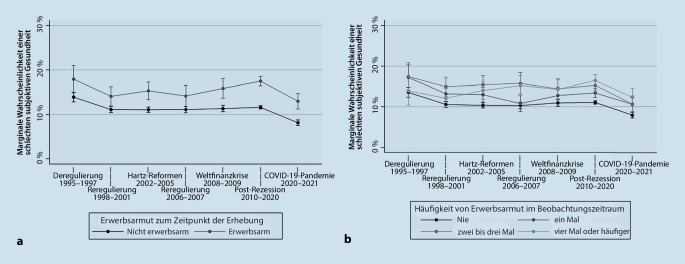

Auch bei den weiblichen Beschäftigten hing die Erwerbsarmut zu allen Zeitphasen signifikant mit einer unzureichenden Gesundheit zusammen (Tab. 3). Die Beziehung zwischen Erwerbsarmut und einer unzureichenden Gesundheit hat im Zeitverlauf für Frauen stetig zugenommen und war in der Phase der COVID-19-Pandemie am stärksten ausgeprägt. Ungeachtet dessen lassen sich keine signifikanten Unterschiede in der Beziehung zwischen subjektiver Gesundheit und Erwerbsarmut zwischen den einzelnen Zeitphasen feststellen. Ähnlich, aber im Vergleich zu Männern geringer, nimmt auch der Zusammenhang zwischen der Häufigkeit von Erwerbsarmut und einer unzureichenden subjektiven Gesundheit im Zeitverlauf zu. Diese ist wiederum in der Phase der COVID-19-Pandemie am stärksten und äußert sich in einem Gradienten in der Gesundheit zuungunsten von Frauen, die im Beobachtungszeitraum häufiger erwerbsarm waren (Tab. 3). Schließlich wird aus Abb. 4a ersichtlich, dass im Verlauf der COVID-19-Pandemie weitaus weniger Frauen ihre subjektive Gesundheit als unzureichend empfanden als in den vorangegangen Zeitphasen. Darüber hinaus zeigt Abb. 4b eine leichte Annäherung der absoluten Gesundheitsunterschiede nach der Häufigkeit von Erwerbsarmut.

## Diskussion

Die vorliegende Studie untersuchte als erste für Deutschland, ob und inwieweit sich der Zusammenhang zwischen subjektiver Gesundheit und Erwerbsarmut im Zuge der COVID-19-Pandemie verändert hat. Hierzu wurde eine Zeitverlaufsperspektive gewählt, welche die Phase der COVID-19-Pandemie in Bezug zu früheren Phasen ökonomischer Krisen und arbeitsmarktpolitischer Reformen setzte. Die Ergebnisse verdeutlichen eine starke Verbesserung der subjektiven Gesundheit im Zuge der COVID-19-Pandemie für beide Geschlechter. Die Erwerbsarmut war im gesamten Zeitraum signifikant mit einer unzureichenden Gesundheit assoziiert, veränderte sich im Zeitverlauf jedoch nicht signifikant. Insbesondere Beschäftigte, die im Zeitverlauf häufiger von Erwerbsarmut betroffen waren, zeigten das höchste Risiko einer unzureichenden Gesundheit. Dieser Zusammenhang nahm im gesamten Beobachtungszeitraum zu und erreichte für beide Geschlechter seinen Höhepunkt in der COVID-19-Pandemie.

Die allgemeine Verbesserung der subjektiven Gesundheit im Zuge der COVID-19-Pandemie konnte auch in anderen Studien belegt werden [43–45]. So verbesserte sich beispielsweise auch in einer niederländischen Längsschnittstudie von van der Weijer et al. [43] die subjektive Gesundheit während der Pandemie. Diese Verbesserung wird nicht auf einen tatsächlichen Anstieg des gesundheitlichen Befindens zurückgeführt, sondern auf ein verändertes Einschätzungsverhalten in Zeiten gesundheitlicher Krisen. Es wird angenommen, dass nicht-infizierte Menschen ihre Gesundheit in Zeiten gesundheitlicher Krisen positiver bewerten als unter normalen Umständen (teilweise auch durch einen sozialen Vergleich mit infizierten Personen). Eine weitere mögliche Erklärung der verbesserten subjektiven Gesundheit liegt in der Pandemie und den damit einhergehenden Herausforderungen. Das Infektionsgeschehen, die Betreuung und das Homeschooling von Kindern oder der Zwang zum Homeoffice verlangen demnach vom Individuum gewisse gesundheitliche Kapazitäten und fördern aus diesem Grund auch einen acht- und pflegsamen Umgang mit der eigenen Gesundheit [46]. Darüber hinaus wird gemutmaßt, dass durch die getroffenen Maßnahmen, wie Homeoffice und Kurzarbeit, zumindest kurzfristig eine stressreduzierende Entschleunigung in der Arbeits- und Lebenswelt stattgefunden hat [47]. Entsprechend zeichnen Studien zur Veränderung der psychischen Gesundheit während der COVID-19-Pandemie ein uneinheitliches Bild und plädieren für eine stärker differenzierte Betrachtung der verschiedenen Belastungslagen entlang spezifischer Subgruppen [48]. Aus diesem Grund ist es nicht nur notwendig, alternative Indikatoren für die Gesundheit, wie beispielsweise das affektive Wohlbefinden, in weiteren Studien miteinzubeziehen, sondern auch die Analysen auf spezifische Subgruppen auszuweiten.

Die Erwerbsarmut hat im Zeitverlauf deutlich zugenommen. Waren zwischen 1995 und 1997 noch ca. 10 % der weiblichen und männlichen Beschäftigten erwerbsarm, waren es in der COVID-19-Pandemie bereits ca. 15 %. Ungeachtet dieses Anstiegs blieb der Zusammenhang zwischen Erwerbsarmut und einer unzureichenden Gesundheit statistisch gesehen konstant und stieg in der COVID-19-Pandemie nur für Frauen leicht an. Diese Ergebnisse reihen sich in Studien der gesundheitlichen Ungleichheitsforschung ein, die im Zeitverlauf konstante Ungleichheiten in der Gesundheit nachgewiesen haben [49]. Dass sich die gesundheitlichen Unterschiede im Zeitverlauf nur leicht verändert haben, weist darauf hin, wie stark die Gesundheit und deren Determinanten in unserer Sozialstruktur verankert und zumindest auf den ersten Blick gegenüber Krisensituationen resilient sind [50].

Auf der anderen Seite können jedoch auch methodische Limitationen in der Erfassung erwerbsarmer Lagen dazu beigetragen haben, dass sich die gesundheitlichen Unterschiede zwischen Erwerbsarmen und Nicht-Erwerbsarmen im Zeitverlauf nur leicht verändert haben. So kann eine geringere Teilnahmebereitschaft sozial benachteiligter Gruppen vor dem Hintergrund pandemiebedingter Herausforderungen dazu beigetragen haben, dass die gesundheitlichen Unterschiede in der COVID-19-Pandemie geringer ausfallen als angenommen [51]. Hinzu kommt, dass Beschäftigte im Niedriglohnbereich wie Minijobber*innen und Leiharbeiter*innen in der Pandemie häufiger von Arbeitslosigkeit betroffen waren und somit nicht Teil der Analysen waren [52, 53]. Der daraus resultierende Selektionseffekt der Pandemie auf die Armutsstruktur der Beschäftigten in Deutschland erschwert zusätzlich einen zeitlichen Vergleich der gesundheitlichen Unterschiede erwerbsarmer und nicht-erwerbsarmer Gruppen.

Daran anknüpfend kann der zum Teil erhebliche Anstieg der Erwerbsarmut während der COVID-19-Pandemie ein Indiz dafür sein, dass mit der Pandemie mehr Beschäftigte kurzfristig erwerbsarm geworden sind. Diese kurzfristige Passage von Erwerbsarmut muss nicht zwangsläufig zu gesundheitlichen Einschränkungen führen, wenn alternative Ressourcen zum Erhalt des Lebensstandards zur Verfügung stehen. Darauf deuten auch die vorliegenden Ergebnisse zur gesundheitlichen Lage von Beschäftigten hin, die im Beobachtungszeitraum häufiger von Erwerbsarmut betroffen waren. So weisen die Ergebnisse nicht nur auf einen Gradienten in der Gesundheit zuungunsten derer hin, die im Zeitverlauf häufiger von Erwerbsarmut betroffen waren. Auch nahm dieser Gradient im Zeitverlauf stetig zu und war für beide Geschlechter in der COVID-19-Pandemie am stärksten ausgeprägt. Gemäß Ressourcentheorie scheinen daher Beschäftigte, die in ihrer Erwerbsbiografie häufiger von Erwerbsarmut betroffen waren, gegenüber Krisensituationen vulnerabler zu sein, gerade da ihnen zur Krisenbewältigung weniger Ressourcen zur Verfügung standen als Personen, die nie oder weniger häufig erwerbsarm waren. Die COVID-19-Pandemie gilt dabei als besondere Krisensituation, da sie die Betroffenen nicht nur vor wirtschaftliche, sondern auch vor gesundheitliche, soziale und familiäre Herausforderungen gestellt hat [54].

Die Beziehung zwischen Erwerbsarmut und einer unzureichenden Gesundheit war bei Männern nicht signifikant, tendenziell aber stärker ausgeprägt als bei Frauen. Dieser tendenziell ausgeprägtere Unterschied in der Gesundheit bei Männern kann ein Indiz dafür sein, dass Männer die Erwerbsarmut als gesundheitlich stärker belastend erleben als Frauen, unter anderem da sie möglicherweise dem gesellschaftlichen Rollenbild eines Alleinernährers nicht entsprechen und vom – bei Männern dominierenden – Normalarbeitsverhältnis abweichen [16, 17]. Jedoch zeichnen die Ergebnisse ein Bild gesellschaftlich fest verankerter Mechanismen der Beziehung zwischen Erwerbsarmut und Gesundheit, die ungeachtet des Geschlechts wirksam sind.

### Limitationen

Trotz der umfangreichen und qualitativ hochwertigen Datenverfügbarkeit des SOEP, die erst eine Zeitvergleichsanalyse des Zusammenhangs zwischen Erwerbsarmut und Gesundheit ermöglichte, weist die vorliegende Untersuchung einige Einschränkungen auf.

Erstens umfasst die SOEP-CoV-Stichprobe nur einen Teil der SOEP-Stichprobe, so dass eine Vergleichbarkeit der Stichproben aufgrund systematischer Ausfälle in den Sondererhebungen von SOEP-CoV eingeschränkt sein kann. Insbesondere Personen mit gesundheitlichen Einschränkungen oder Erwerbsarme könnten aufgrund der pandemischen Lage und finanzieller Unsicherheiten nicht an der SOEP-CoV-Befragung teilgenommen haben. Dadurch können Ungleichheiten in der Gesundheit unterschätzt worden sein. Zusätzliche Analysen, die ausschließlich nur solche Befragungspersonen beinhalteten, die sowohl am SOEP als auch an SOEP-CoV teilgenommen haben, zeigen jedoch keine substanziellen statistischen Abweichungen. Auch die Anwendung von Gewichten, die Designeffekte und Nonresponse berücksichtigen und eine Verallgemeinerung auf die Gesamtpopulation in Deutschland zulassen, führte zu keiner substanziellen Veränderung der Ergebnisse. Auf die Anwendung einer Gewichtung wurde in der vorliegenden Studie verzichtet, da diese für die zweite Sondererhebung von SOEP-CoV nicht zur Verfügung stand.

Zweitens wurde im Gegensatz zur normalen Befragung im SOEP die Sondererhebung von SOEP-CoV ausschließlich telefonisch durchgeführt. Diese Veränderung in der Erhebungsmethode kann einen Einfluss auf das Antwortverhalten der Befragten gehabt haben und damit die Vergleichbarkeit der Ergebnisse im Zeitverlauf zusätzlich einschränken. Insgesamt wird der Einfluss der veränderten Erhebungsmethode aber als gering eingestuft, da die Veränderungen in der subjektiven Gesundheit bedeutsam und so groß waren, dass sie nicht allein durch eine veränderte Erhebungsmethode erklärt werden können.

Drittens erfassen das SOEP und SOEP-CoV keine umfassenden Informationen zum gesundheitlichen Befinden, zur Qualität der ausgeführten Tätigkeit und zum aktuellen Lebensstandard. So erfassen die Sondererhebungen von SOEP-CoV wesentliche Indikatoren zur Erklärung der Beziehung zwischen Erwerbsarmut und Gesundheit nicht, wie beispielsweise den aktuellen Lebensstandard, Gratifikationskrisen oder die Sorge vor einem Arbeitsplatzverlust. Analysen zu sich verändernden Erklärungsmechanismen konnten daher nicht durchgeführt werden.

Viertens können wir aufgrund des gewählten Querschnittsdesigns und der durchgeführten Analysen keine kausalen Schlüsse zum Zusammenhang zwischen subjektiver Gesundheit und Erwerbsarmut ziehen. Existierende Studien messen aber dem Wirken von Erwerbsarmut auf Gesundheit eine höhere Bedeutung bei als dem gegensätzlichen Wirkungspfad [55].

Schließlich ermöglicht die Einordnung der Erhebungswellen in unterschiedliche Phasen ökonomischer Krisen und arbeitsmarktpolitischer Reformen zwar eine bessere Übersicht und eine zeitliche Kontextualisierung der Geschehnisse. Diese darf aber nicht darüber hinwegtäuschen, dass insbesondere arbeitsmarktpolitische Reformen zeitlich oftmals in andere, hier erfasste Phasen hineinwirken. Insbesondere die Hartz-IV-Reformen und die damit verbundenen Deregulierungsmaßnahmen werden zumindest als weiterer Grund dafür diskutiert, weshalb sich Deutschland gegenüber jüngeren Krisen als relativ widerstandsfähig erwiesen hat [56]. Darüber hinaus variieren Arbeitsmarktreformen in ihrem Umfang und Wirken auf den Arbeitsmarkt und werden oftmals von weiteren Maßnahmen der Haushalts‑, Sozial‑, Familien- und Rentenpolitik flankiert. Diese können in ihrem Zusammenspiel unterschiedliche Einflüsse auf die soziale Lage von Beschäftigten, Haushaltsmitgliedern und spezifischen Subgruppen haben, die – wenn überhaupt möglich – einer differenzierten Untersuchung bedürfen.

## Fazit

Trotz einer verbesserten subjektiven Gesundheit im Zuge der COVID-19-Pandemie in Deutschland zeigen sich im Beobachtungszeitraum von 27 Jahren weitestgehend persistente Unterschiede in der Gesundheit zuungunsten Erwerbsarmer. Der Zusammenhang zwischen Erwerbsarmut und Gesundheit hat in der COVID-19-Pandemie nur leicht zugenommen und dies insbesondere für Beschäftigte, die im gesamten Beobachtungszeitraum häufiger von Erwerbsarmut betroffen waren. Dies verdeutlicht einerseits die feste gesellschaftliche Verankerung von Erwerbsarmut als Determinante einer unzureichenden Gesundheit. Andererseits kann der leichte Anstieg der gesundheitlichen Unterschiede zuungunsten Erwerbsarmer dahingehend gedeutet werden, dass die COVID-19-Pandemie, anders als vorangegangene Krisen und Umbrüche, nicht nur mit ökonomischen Herausforderungen einherging, sondern die Betroffenen auch vor gesundheitliche, soziale und familiäre Herausforderungen gestellt hat. Insbesondere Erwerbsarme gelten hierbei als besonders vulnerable Gruppe, da sie nur über ein geringes Maß an Ressourcen verfügen, um adäquat auf die unterschiedlichen Herausforderungen der Pandemie reagieren zu können.
